# Prednisone Reprograms the Transcriptional Immune Cell Landscape in CNS Autoimmune Disease

**DOI:** 10.3389/fimmu.2021.739605

**Published:** 2021-08-13

**Authors:** He Li, Yuehan Gao, Lihui Xie, Rong Wang, Runping Duan, Zhaohuai Li, Binyao Chen, Lei Zhu, Xianggui Wang, Wenru Su

**Affiliations:** ^1^State Key Laboratory of Ophthalmology, Zhongshan Ophthalmic Center, Sun Yat-sen University, Guangzhou, China; ^2^Eye Center of Xiangya Hospital, Central South University, Changsha, China; ^3^Hunan Key Laboratory of Ophthalmology, Xiangya Hospital, Central South University, Changsha, China

**Keywords:** prednisone, glucocorticoids, autoimmune uveitis, single-cell RNA sequencing, CNS autoimmune diseases

## Abstract

Glucocorticoids (GCs) are widely used immunosuppressive drugs for autoimmune diseases, although considerable gaps exist between current knowledge of the mechanisms of GCs and their conclusive immune-regulatory effects. Here we generated a single-cell transcriptional immune cell atlas based on prednisone-treated or untreated experimental autoimmune uveitis (EAU) mice. Immune cells were globally activated in EAU, and prednisone partially reversed this effect in terms of cell composition, gene expression, transcription factor regulation, and cell-cell communication. Prednisone exerted considerable rescue effects on T and B cells and increased the proportion of neutrophils. Besides commonly regulated transcriptional factors (Fosb, Jun, Jund), several genes were only regulated in certain cell types (e.g. Cxcr4 and Bhlhe40 in T cells), suggesting cell-type-dependent immunosuppressive properties of GC. These findings provide new insights into the mechanisms behind the properties and cell-specific effects of GCs and can potentially benefit immunoregulatory therapy development.

## Introduction

Despite the expansion of novel immunosuppressants in the recent decades, glucocorticoids (GCs) remain the most widely used anti-inflammatory and immunosuppressive agents in clinical medicine. They are indispensable for achieving rapid disease control, especially of severe autoimmune diseases (1–4). However, the use of GCs is mostly based on historical experiences and knowledge from case series. For decades, the clinical application of GCs has exceeded our current knowledge of their immunosuppressive effects. Thus, there is an urgent need for research on the regulatory properties of GCs and their effects on the immune system.

Cytosolic GC receptors serve as the main mediators of the immunosuppressive and anti-inflammatory effects of GCs (5). Upon GC binding, they translocate into the nucleus, activate the expression of anti-inflammatory proteins, and inhibit the production of pro-inflammatory proteins (2). Recent studies have also indicated other potential pathways associated with GCs, such as the regulatory properties of GC receptors without GC binding and even the receptor-independent effects of GCs (2, 6). Moreover, growing evidence suggests that the underlying mechanisms of GCs are cell type-dependent (7, 8). Due to this complexity, it is necessary to comprehensively depict the effects of GCs on the immune system from multiple perspectives and characterize the transcriptional modifications across different immune cell subpopulations.

Autoimmune diseases mainly affecting the central nervous system (CNS) include multiple types of diseases and affect millions of people (9). These diseases usually involve responses to self-antigen exposure and aberrant activation of the immune system. They are also chronic, painful, debilitating, and potentially life-threatening, thereby significantly affecting the quality of life. Among them, autoimmune uveitis, which mainly affects the vascular organs of the eye (iris, ciliary body, and choroid), is considered one of the principal causes of preventable blindness worldwide (10). It not only presents as an isolated entity or a part of a systemic autoimmune disease affecting CNS, but also allows direct observation of the inflammatory condition of the central nervous system, serving as a satisfactory model for CNS autoimmune disease studies. Moreover, GCs serve as the first-line systemic treatment for patients with sight-threatening uveitis, despite the lack of a comprehensive and in-depth understanding of their immunosuppressive properties (4, 10, 11).

To explore the effect of GCs on the immune system in the context of CNS autoimmune states, we developed experimental autoimmune uveitis (EAU) mouse models and performed single-cell profiling of the lymph node transcriptome (12, 13). We analyzed approximately 35,000 single cells of lymph nodes sampled from normal and EAU mice without additional treatment or prednisone. We investigated the effects of EAU and prednisone on the mouse immune system in the context of cell type composition, cell type-specific gene expression, transcription factor (TF) regulation, and intercellular communication. These showed widespread alterations in EAU, most strongly affecting CD4+ T cells. Interestingly, prednisone partly countered such alterations. Our results not only support the well-established participation of T cells and monocytes/macrophages in the onset of uveitis and effects of prednisone, but also comprehensively reveal the transcriptional modifications of the immune cell subsets at the single-cell level.

## Materials and Methods

### EAU Model Induction and EAU Clinical Score

To induce EAU, the mice were injected subcutaneously with an emulsion, consisting of 2 mg/mL of human IRBP1-20 (GPTHLFQPSLVLDMAKVLLD, GiL Biochem, Shanghai, China) and complete Freund’s adjuvant (BD Difco, San Jose, CA, USA) containing 2.5 mg of Mycobacterium tuberculosis strain H37Ra (BD Difco, San Jose, CA, USA) in a 1:1 volume ratio, were injected to mice (13). Additionally, 0.25 µg pertussis toxin (List Biological Laboratories, Campbell, California, USA) was dissolved in PBS, and injected on the same day and 2 days after immunization.

Funduscopic examination of EAU progress with the Micron IV fundus camera (Phoenix Co., Campbell, CA, USA) was performed, and the clinical findings were graded from 0 to 4 scoring based on observable infiltration and vasculitis in the retina (14). The clinical score was assessed in a blinded manner as followed: 0: Normal retina; 0.5/trace: minimal vasculitis; 1: Mild vasculitis; multiple, peripheral, and focal lesions; 2: Severe vasculitis (large size, thick wall, infiltrations); diffuse chorioretinal lesions and/or infiltrations; linear lesions; 3: Pattern of linear lesions; large confluent chorioretinal lesions; subretinal hemorrhages; 4: Large retinal detachment.

### Treatment of Mice

Prednisone (Selleck Chemicals, Houston, TX, USA) was dissolved in DMSO (0.1%, Sigma). Mice with EAU induction were orally administered with prednisone (10 mg/kg/day) or vehicle control (0.1% DMSO) for 2 weeks after immunization (15). Each groups included six mice.

### Lymph Node Collection and Single-Cell Suspension Preparation

Cervical dLNs isolated from the neck of mice were isolated into single cells by filtration through a with 20-μm strainer (352235, Falcon). After washing twice with PBS, the cells viability analyzed by Trypan blue in each sample exceeded 85%.

### Flow Cytometric Analysis

After staining with live/dead dye (Thermo Fisher Scientific, Waltham, MA, USA), the cells were stained with surface markers: anti-mouse CD4 (GK1.5, catalog 100434, BioLegend), anti-mouse CD138 (Syndecan-1) (clone 281-2), anti-mouse CXCR5 (clone L138D7), anti-mouse PD-1 (clone 29F.1A12), anti-mouse CD90.2 (Thy-1.2) (clone 53-2.1), anti-human/mouse CD45R (B220) (clone RA3-6B2), anti-mouse F4/80 (clone BM8), anti-mouse CD11c (clone HL3) and analyzed *via* flow cytometry (BD LSRFortessa). After stimulation of 5 ng/ml phorbol myristate acetate (Sigma), 1 μg/ml brefeldin A (Sigma), and 0.5 mg/ml ionomycin (Sigma) at 37°C for 4 hours, and then fixation for 30 minutes and permeabilization for 1 hour at room temperature, the cells were stained with intracellular antibodies for 4°C overnight: anti-mouse IL-17A (TC11-18H10.1, catalog 506930, BioLegend), anti-mouse IFN-γ (XMG1.2, catalog 505808, BioLegend). The results were evaluated with FlowJo software (version 10.0.7, Tree Star, Ashland, OR, USA).

### Single-Cell RNA Sequencing

We mix cells from cervical lymph nodes which from three group (normal, EAU treated with vehicle and EAU treated with prednisone) and each group includes three mice. After that, three mix samples respectively from three groups are used to be sequenced. Before sequencing, we did not select immune cells *via* flow cytometry.

#### scRNA-Seq Data Processing

We used the Chromium Single Cell 5′ Library (10× Genomics chromium platform; Illumina NovaSeq 6000), Gel Bead and Multiplex Kit, and Chip Kit to acquire barcoded scRNA-seq libraries. Preparation of single-cell RNA libraries were performed with the Chromium Single Cell 5′ v2 Reagent (10× Genomics, 120237) kit. The quality of the libraries was tested with FastQC software. Demultiplexing and barcoding of the sequences from the 10× Genomics scRNA-seq platform alignment to the mm10 reference and quantification of sequencing reads for each sample were performed using CellRanger (Version 4.0.2, 10× Genomics) with default parameters.

#### scRNA-Seq Quality Control

For quality control, the Seurat package (version 3.1, https://github.com/satijalab/seurat) was used. Cells were filtered out if they showed greater than 15% of mitochondrial genes and fewer than 300 or greater than 10,000 detected genes. Genes not detected is also not include in analysis.

#### scRNA-Seq Analysis

For the scRNA-seq data analysis, we used Seurat package for normalization, dimensionality reduction, clustering as well as DEG analysis. We log-normalized the data with the ‘NormalizeData()’ before clustering and reduction and scaled the data with the top 2000 most variable genes by using the ‘FindVariableFeatures()’ script. The clustering and dimensionality method were used with the ‘FindClusters()’ at an appropriate resolution to identify significant clusters, which uses a shared nearest neighbor parameter optimized for each combined dataset modularity optimization-based clustering algorithm. 2-t-SNE clustering was performed using the ‘RunTSNE()’ function. DEGs were determined using the ‘FindAllMarkers()’ function. A disease-related DEG dataset was established (p value < 0.05, |Log2 fold-change| > 0.25).

#### GO Analysis

All GO enrichment analysis was performed using Metascape (www.metascape.org) (78) to visualize functional patterns in the gene clusters. Statistical analysis was used for GO and pathway enrichment analyses of the DEGs.

#### Transcription Factor-Target Gene Network Analysis

Based on the gene regulation identified in our scRNA-seq data, we utilized the GENIC3 R packages (version 1.6.0) (16), as well as the RcisTarget database (version 1.4.0) (17) of the SCENIC (version 1.1.2.2) (18) workflow to predict the transcription factor and their downstream genes. We used GENIE3 to computerize the genetic regulatory networks from our expression data, including EAU DEGs, prednisone DEGs or rescue DEGs, for each cell type. We further used RcisTarget databases to recognize the enriched transcription factor-binding motifs and those potential downstream genes (regulons). Figures showed the transcription factor targets with high-confidence annotation, with the Cytoscape software (version 3.7.1) (19).

#### Cell-Cell Communication Analysis

The intercellular communication was predicted with CellPhoneDB software (version 1.1.0) (www.cellphonedb.org) (20). We selected and analyzed the ligand-receptor pairs expressed in at least 10% of cells of a given type. The interaction was considered nonexistent if either the ligand or the receptor was undetectable. We compared the average expression of ligand-receptor pairs in different cell types, and selected pairs with p < 0.05 for further computerization of intercellular communication.

### Statistical Analysis

GraphPad Prism Software was used to data analysis. The values are represented as the mean ± SD. Statistical analysis was performed using an unpaired, two‐tailed Student’s t-test or one-way ANOVA. p values above 0.05 were considered as not significant, NS; *, p < 0.05; **, p < 0.01; ***, p < 0.001; and ****, p < 0.0001.

## Results

### Construction of Lymph Node Single-Cell Atlases of Normal and EAU mice

**Figure 1:**
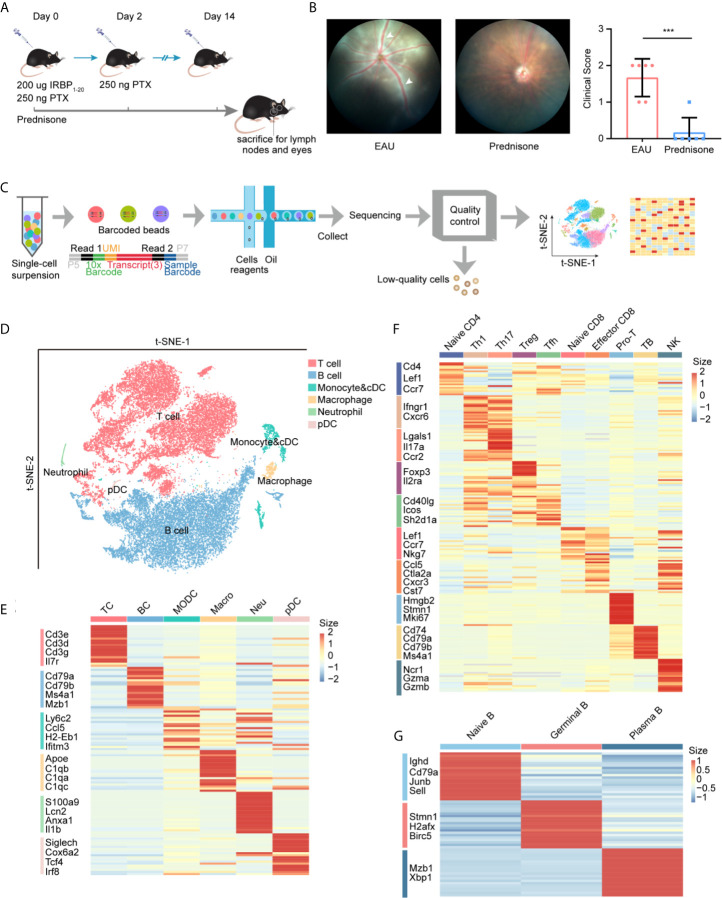
study design and unbiased classification of immune cells by scRNA-seq. **(A)** research design. Cervical lymph nodes of EAU mice, prednisone-treated EAU mice, and normal subjects were studied. Cervical lymph nodes cells which from three group (normal, EAU treated with vehicle and EAU treated with prednisone) and each group includes three mice, were mixed. Three mix samples respectively from three groups are used to be sequenced without selection of immune cells *via* flow cytometry. IRBP, interphotoreceptor retinoid-binding protein. PTX, pertussis toxin. **(B)** Left: fundus photography of EAU and prednisone-treated EAU mice. Right: the clinical scores of EAU or prednisone-treated mice. Each group contains six mice. White Arrows indicate inflammatory exudation and vascular deformation. The P-values are exact two-sided generated by students’ t test. ***p < 0.001. **(C)** Flowchart of the scRNA-seq process. **(D)** t-SNE embedding shows different cell types in lymph nodes. **(E)** Heatmap of marker genes for each type of cells. **(F)** Heatmap of marker genes for subtypes of T cells. **(G)** Heatmap of marker genes for subtypes of B cells.

We first developed EAU mouse models by immunizing mice with the retinal protein interphotoreceptor retinoid-binding protein, and prepared non-treated mice as normal controls (see *Methods*). The EAU mice were evenly matched and randomized into two groups: (1) EAU group with no additional treatment and (2) prednisone group treated with prednisone (**Figure 1A**). After 14 days, the clinical scores of EAU mice were significantly increased compared to those of normal controls, indicating the onset of autoimmune uveitis in the mouse models (**Figure 1B**). In contrast, prednisone significantly reduced the inflammatory symptoms of EAU, as revealed by fundus photography and the clinical scores (**Figure 1B**).

To explore the underlying mechanisms of action of prednisone and the transcriptomes of lymph nodes of EAU mice, we generated single-cell RNA transcriptional profiles of immune cells from normal, EAU, and prednisone-treated EAU mice by isolating the lymph nodes of these mice and performing single-cell RNA sequencing. After the quality control process, we acquired 35000 high-quality immune cells for further analysis (see *Methods*) (**Figure 1C**). Then, we processed the sequencing data through integration, normalization, and clustering. We first identified six major cell types: (1) T cells, (2) B cells, (3) monocytes and conventional dendritic cells (cDCs), (4) macrophages, (5) neutrophils, and (6) plasmacytoid dendritic cells (pDCs), based on their corresponding marker genes (**Figures 1D, E** and **Supplementary Figure 1A**). We further differentiated cDCs and monocytes based on the expressions of *Ly6c2*, *Ccr2*, *Ccl22*, and *Fscn1* (**Supplementary Figures 1B, C**). To analyze T cell subsets in detail, T cells were subdivided into nine subsets with distinct properties (**Figure 1F** and **Supplementary Figures 2A, B**). The Th1 and Tfh clusters were further re-clustered based on the expressions of *Cxcr6* and *Tcf7* (**Supplementary Figures 2C–E**). Additionally, B cells were subdivided into three subpopulations: (1) naïve B cells, (2) germinal B cells, and (3) plasma B cells (**Figure 1G** and **Supplementary Figures 1D, E**).

Taken together, we constructed an integrative transcriptional atlas containing multiple immune cell subpopulations and established a cellular profile to further understand the effects of EAU and prednisone on the immune system.

### Prednisone Affects Cell Type Composition

**Figure 2:**
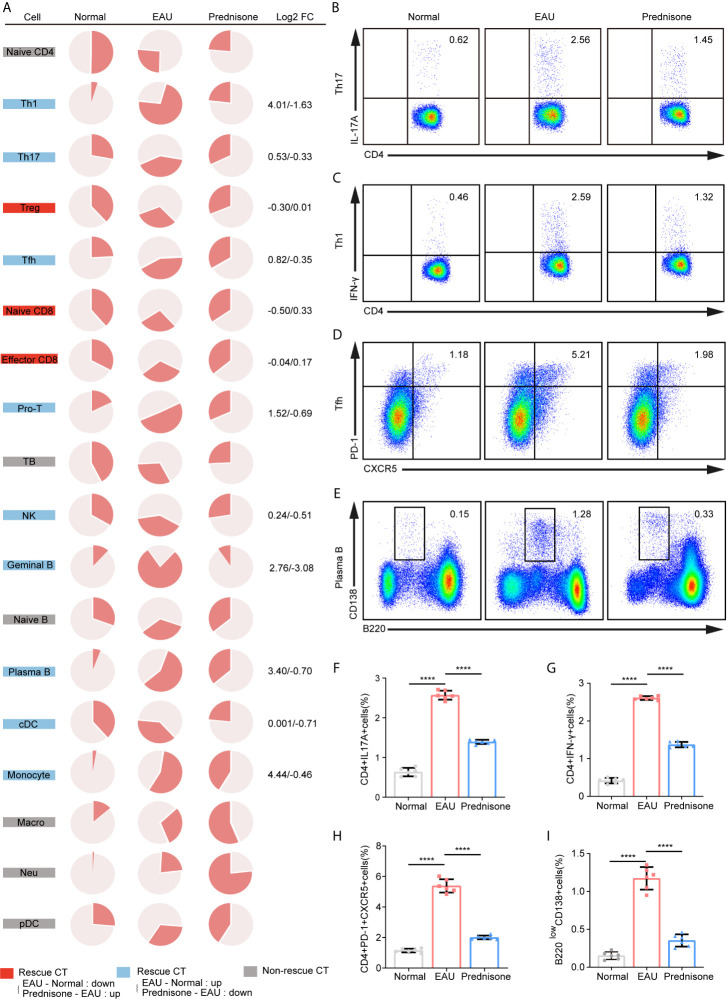
The proportions of immune cells of EAU and prednisone-treated mice. **(A)** Pie charts of proportions of each type of cells in three group. The Log2FC values of the proportion were shown on the right. Normal, normal subject; EAU, experimental autoimmune uveitis mouse group; Prednisone, prednisone-treated mouse group. **(B–E)** Flow cytometry indicates the proportion of Th17 **(B)**, Th1 **(C)**, Tfh **(D)** and plasma B cells **(E)**, which was performed once with a cohort of 6 mice. **(F–I)** Bar plots show the proportions of Th17 **(F)**, Th1 **(G)**, Tfh **(H)** and plasma B cells **(I)** in normal controls, EAU mice treated with vehicle, and prednisone-treated EAU mice. Each group contains six mice. The P-values are exact two-sided generated by students’ t test. ****p < 0.0001.

To depict the alterations in cell type composition in EAU and due to prednisone treatment, we investigated the changes in cell type proportions in healthy, EAU, and prednisone-treated EAU mice (**Figure 2A**). In EAU mice, the proportions of Th1, Th17, Tfh, and proliferating T cells were increased, while the proportions of Treg cells and two CD8+T cell subsets were reduced (**Figure 2A**). B cell subsets were assumed to participate in disease progression, since the proportions of germinal and plasma B cells were also increased in EAU mice (**Figure 2A**). The proportions of myeloid leukocytes, including monocytes, pDCs, macrophages, and neutrophils, were also higher in EAU mice than in normal controls.

In the prednisone group, we observed widespread rescue effects of prednisone on nearly all immune cell types, especially the lymphocyte subpopulations (**Figure 2A**). These rescue effects were highly evident among CD4+T cells, particularly Th1, Th17, and Tfh cells, indicating that prednisone may inhibit the differentiation of Th1, Th17, and Tfh cells (**Figure 2A**). Furthermore, we validated the changes in the proportions of these three cell types using flow cytometry (**Figures 2B–D**). We observed profound rescue effects of prednisone on Th1, Th17, and Tfh cells, consistent with the previous findings (**Figures 2F–H**). The alterations in the proportions of germinal and plasma B cells were reversed using prednisone treatment, suggesting that prednisone influences both humoral and cellular immunity to achieve its clinical benefit (**Figure 2A**). The proportion of plasma B cells was further validated using flow cytometry (**Figures 2E, I**).

### Prednisone Reverses EAU-Associated Gene Expression Alteration in Lymph Nodes

**Figure 3:**
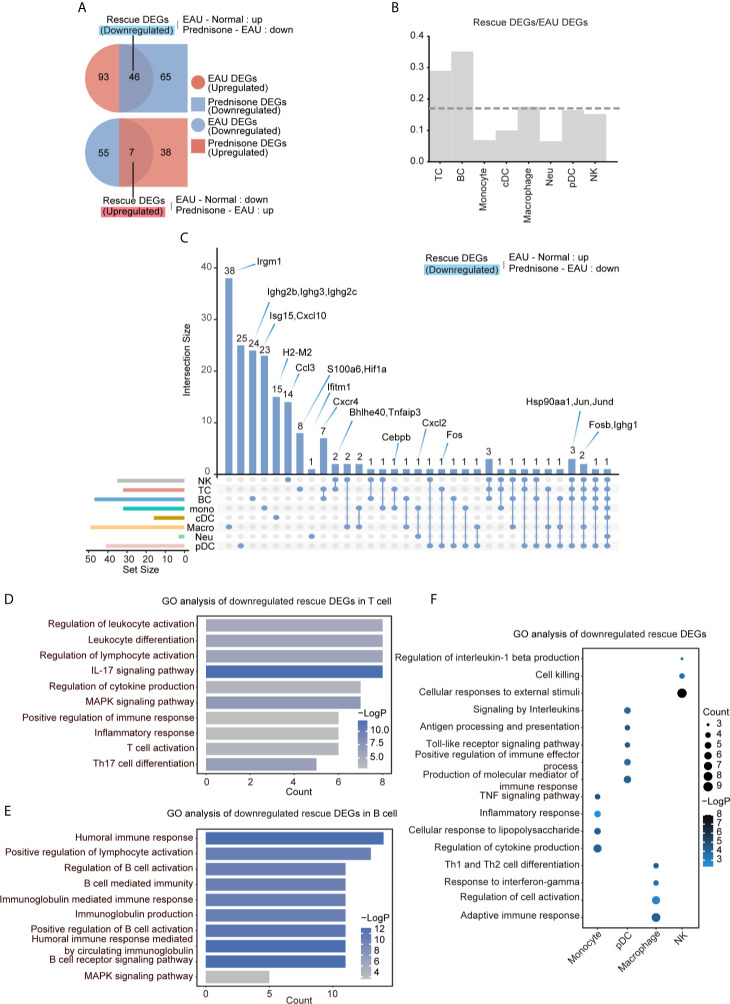
Prednisone partly reversed the EAU-associated gene expression. **(A)** Venn diagrams of the numbers of EAU, prednisone and rescue DEGs. The overlapping parts indicate the numbers of downregulated rescue DEGs (top) and upregulated rescue DEGs (bottom). **(B)** Bar plots of the proportion of rescue DEGs compared to EAU DEGs. The dotted line indicates average proportion of rescue DEGs/EAU DEGs. **(C)** Venn diagram shows the downregulated DEGs in each cell types. Key genes were labeled on the top. **(D–F)** GO analysis of downregulated rescue DEGs in T cells **(D)**, B cells **(E)**, monocytes, pDCs, macrophages and NK cells **(F)**.

To delineate the transcriptional changes caused by EAU and prednisone, we identified differentially expressed genes (DEGs) between normal and EAU mice (EAU DEGs) and between EAU and prednisone-treated EAU mice (prednisone DEGs) (**Figure 3A**). Based on these genes, we further distinguished “rescue DEGs”, which refer to EAU DEGs that exhibited opposite regulatory tendencies after prednisone treatment (**Figure 3A**). We found that prednisone exerted considerable influence on the immune system of EAU mice, with 46 out of 139 upregulated EAU DEGs and 7 out of 62 downregulated EAU DEGs rescued by prednisone (**Figure 3A**).

To characterize the cell type-specific gene regulation profiles, we attributed EAU and rescue DEGs to eight major cell types: (1) T cells, (2) B cells, (3) natural killer (NK) cells, (4) monocytes, (5) cDCs, (6) pDCs, (7) macrophages, and (8) neutrophils (**Figure 3B**). T and B cells were the most strongly affected subsets based on their high rescue DEGs-to-EAU DEGs ratios (**Figure 3B**). Macrophages, pDCs, and NK cells, which may also serve as targets of prednisone, exhibited slightly smaller rescue DEGs-to-EAU DEGs ratios compared to those of T and B cells (**Figure 3B**).

To further explore the possible mechanisms of the immunosuppressive properties of prednisone, we analyzed the commonly and specifically rescued genes in the eight cell types, as shown in the Venn diagram (**Figure 3C**). The most commonly rescued genes were those that encode activator protein 1 (AP-1) transcription factor components, including *Fosb* (freq = 5), *Fos* (freq = 2), *Jun* (freq = 4), and *Jund* (freq = 4), and heat shock protein components, particularly *Hsp90aa1* (freq = 4) (**Figure 3C** and **Supplementary Figure 4A**). Glucocorticoids have been reported to downregulate the expression of several AP-1 components and reduce the DNA-binding ability of the AP-1 components to their cognate DNA motifs (1). Overall, these factors may play central roles in the immunosuppressive properties of prednisone to inhibit highly pro-inflammatory transcriptional signatures across different immune cell subsets in EAU.

In addition to widely regulated genes, we identified genes that were specifically rescued in different cell subsets. We found that *Cxcr4*, which plays a central role in cell homing and retention, was rescued in both T and B cells (21, 22). *Hif1a* was specifically rescued in T cells, while several *Ighg* family members were rescued in B cells. As for the genes in the non-lymphoid cells, both *Cxcl2* (rescued in monocytes and neutrophils) and *Cxcl10* (rescued only in monocytes) possibly contributed to the progression of uveitis by recruiting T cells and DCs (**Figure 3C**). Interestingly, in macrophages, we identified a specifically rescued gene, *Irgm1*, which is an important molecular regulator that promotes the disruption of the blood-brain barrier to initiate inflammation (23) (**Figure 3C**).

To better understand the effects of EAU and prednisone in the context of potential cellular functions, we performed Gene Ontology (GO) analysis on EAU, prednisone, and rescue DEGs in different cell subsets (**Figures 3D–F** and **Supplementary Figures 3A–D**). In EAU, T cells and monocytes exhibited significant alterations towards the “activation” side (**Supplementary Figure 3A**). Moreover, cDCs and B cells may participate in several immune processes in EAU, as they showed upregulated expressions of genes involved in inflammation, cytokine production, antigen presentation, and cell proliferation (**Supplementary Figure 3A**). Upon prednisone treatment, the activation of T cells and monocytes was robustly suppressed, with enrichment in “regulation of cytokine production” and “lymphocyte activation” (in T cells), as well as “myeloid leukocyte migration”, “leukocyte chemotaxis” and “inflammatory response” (in monocytes) (**Supplementary Figure 3B**).

Regarding the rescue effect of prednisone on T cells, we identified categories closely associated with Th17, including “IL-17 signaling pathway”, “Th17 cell differentiation”, “proinflammatory lymphocyte activation”, and “inflammatory response” (**Figure 3D**). We not only identified these categories, which were globally rescued in CD4+ and CD8+ T cells, but also found specifically rescued categories in CD4+ T cells, including “apoptotic signaling pathway” and “HSP90 chaperone cycle for steroid hormone receptors” (**Supplementary Figure 3C**). More specifically, the category “IL-17 signaling pathway” was rescued in Th17 cells, while “MAPK signaling pathway” and “inflammatory response” were rescued in Tfh cells (**Supplementary Figure 3D**). The rescued effect of prednisone on B cells was associated with “humoral immune response”, “B cell-mediated immunity” and “regulation of B cell activation” (**Figure 3E**). In addition, the rescued DEG of myeloid leukocytes showed functional enrichment of “inflammatory response” (monocytes), “regulation of cell activation” (macrophages), “antigen processing and presentation” (pDC), and “cell killing” (NK cells) (**Figure 3F**).

### EAU and Prednisone Affect Cell Type-Specific Gene Expression

**Figure 4:**
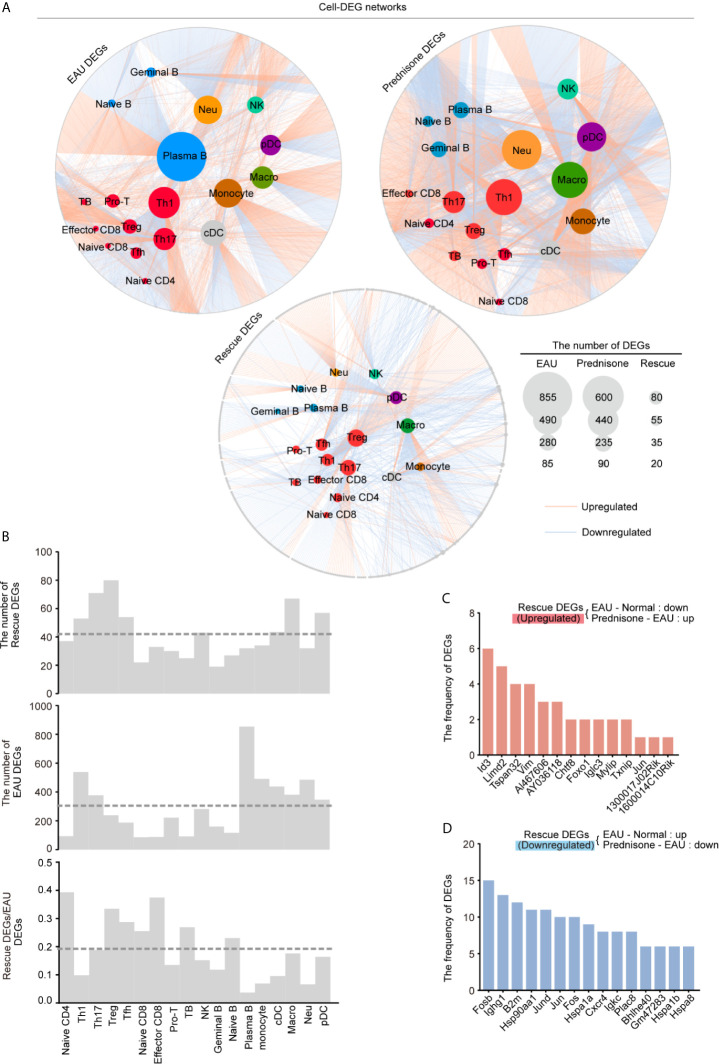
Gene expression networks show cell-type-specific regulation among different cell types. **(A)** Network plots show the EAU, prednisone and rescue DEGs in each types of cells. The color of the internal nodes indicates cell types, and the gray edge represents DEGs. The lines between them attribute the DEGs to each cell types. **(B)** Bar plots show the number of rescue DEGs and EAU DEGs, and the rescue/EAU DEGs in each subtypes. The dotted line indicates average proportion of rescue DEGs/EAU DEGs. **(C, D)** Bar plots indicate the frequency of upregulated **(C)** and downregulated **(D)** rescue DEGs.

To accurately explore the cell type-specific EAU, prednisone, and rescue DEGs, we further classified the DEGs into subpopulations defined in each cell type, generating gene regulatory networks across 18 types of immune cells. In **Figure 4A**, the sizes of the subpopulation circles suggest that both EAU and prednisone exhibit cell type-specific gene regulation effects.

In EAU, gene expression in plasma B cells was most strongly affected, suggesting the spontaneous production of autoantibodies. As expected, Th1 and Th17 cells were the top two T cell subsets influenced by EAU, indicating the central role of activated CD4+T cells in EAU (**Figures 4A, B**) (24). Autoimmunity also had a considerable effect on myeloid leukocytes, including monocytes, macrophages, neutrophils, and DCs (**Figure 4A**). Moreover, prednisone alone significantly affected myeloid cells (monocytes, macrophages, neutrophils, and pDCs) and T cells. Not surprisingly, Th1, Th17, and Treg cells were the top three affected T cell subsets based on their large DEG numbers.

Strikingly, we identified the largest number of DEGs in the Treg subpopulation, although the numbers of EAU DEGs and prednisone DEGs of Treg cells were relatively small compared to those of other cell subsets (**Figure 4A**). The rescue DEGs-to-EAU DEGs ratio further supported this observation, indicating an unexpected rescue effect of prednisone on Treg cells (**Figure 4B**). We also observed a considerable rescue effect on Th17 and Tfh cells and an unexpectedly strong rescue effect in naïve CD4+ T cells, naïve CD8+ T cells, and effector CD8+ T cells (**Figure 4B**). In contrast, given the relatively large numbers of EAU and prednisone DEGs in B cells and myeloid cell subsets, the rescue effect seemed weak in these cell subsets, suggesting that the underlying mechanisms of action of prednisone may not involve directly countering the effects of EAU, but distinctly regulating these types of cells (**Figure 4B**).

Notably, *Id3*, *Limd2*, Tspan32, and *Vim* were downregulated in EAU and upregulated by prednisone in more than four cell types (**Figure 4C**). The rescued genes that were upregulated in EAU and downregulated by prednisone included genes encoding AP-1 components (*Fosb*, *Jund*, *Jun*, and Fos), immunoglobulins (*Ighg1* and *Igkc*), heat shock proteins (*Hsp9011a*, *Hspa1a*, *Hspa1b*, and *Hspa8*), and chemokine receptors (*Cxcr4*) (**Figure 4D**). Overall, these results provide a global assessment of the effects of EAU and prednisone on the immune system of mice at the single-cell level.

### EAU and Prednisone Reprogram Transcriptional Regulatory Networks

**Figure 5:**
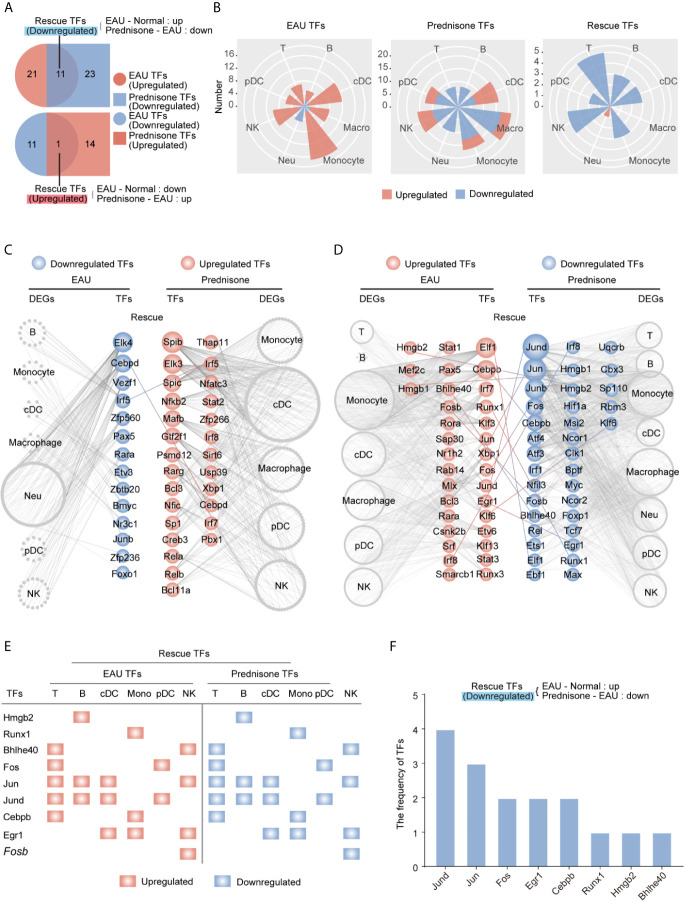
Changes of the transcription factors of EAU and prednisone-treated EAU mice. **(A)** Venn diagrams show the numbers of EAU, prednisone and rescue TFs. The overlapping parts indicate the numbers of upregulated or downregulated TFs. **(B)** Rose diagrams show the numbers of EAU, prednisone and rescue DEGs in each cell types. **(C, D)** Networks show the downregulated **(C)** and upregulated **(D)** EAU or prednisone TFs in each cell types. The circles indicate TFs. The gray edge indicates the downstream DEGs. The line between them indicates rescue TFs. **(E)** The upregulated rescue TFs in each cell types. **(F)** Bar plots indicate the frequency of downregulated rescue TFs.

Given the strong effect of EAU and prednisone on TFs as previously shown, we applied SCENIC to the transcriptional atlases and determined the critical TFs regulating EAU and prednisone DEGs in different cells types. Similarly, we defined “rescue TFs” as the TFs that were dysregulated in EAU and rescued by prednisone (**Figure 5A**). Interestingly, autoimmunity had a significant impact on mainly monocytes and cDCs, while prednisone alone had a widespread effect on nearly all cell types (**Figure 5B**). Although both the numbers of EAU and prednisone TFs were relatively small in T and B cells, both cell types were significantly influenced by rescue effects, which were likely due to the anti-inflammatory effects of prednisone on transcriptional regulatory networks (**Figure 5B**).

Furthermore, TFs regulated by EAU and prednisone were attributed to different types of immune cells, along with their theoretical downstream DEGs (**Figures 5C, D**). Our results showed that rescued TFs included AP-1 components (*Jund, Jun, Junb*, and *Fos*), leucine zipper (*Cebpb* and *Cebpd*), interferon regulatory factors (*Irf1, Irf5*, and *Irf8*), and amphoterin elements (*Hmgb1* and *Hmgb2*) (**Figures 5C, D**). The central roles of the AP-1 family are further indicated in **Figures 5E, F**, where two AP-1 components (*Jund* and *Jun*) not only appeared most frequently in the rescue list, but were also rescued in T cells, B cells, and DCs, which are essential in the pathogenesis of uveitis. In addition, the TFs *Hmgb2*, *Bhlhe40*, and *Cebpb* rescued in T and B cells may also play important roles in prednisone treatment. Focusing on the central CD4+ T cell subsets (Tfh, Th17, and Treg cells), we further identified TFs that may play key roles in both the effects of EAU and prednisone. In addition to AP-1 members, *Cebpb* and *Hif1a* were rescued in all three subsets. These findings provide evidence for the mechanisms of EAU pathogenesis and prednisone treatment (**Supplementary Figure B**).

Taken together, these data reveal that EAU-associated TF regulatory networks were strongly affected by prednisone and that AP-1 components were the TFs most frequently affected by EAU and prednisone.

### Prednisone Alleviates Aberrant Intercellular Communication in EAU

**Figure 6:**
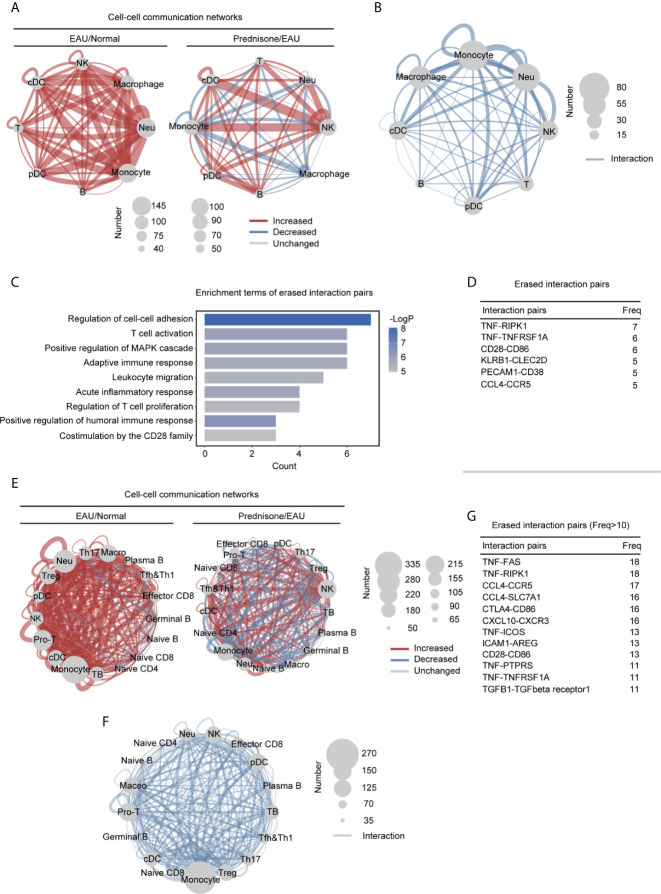
Alteration of the intercellular interactions between different cell types in EAU and prednisone-treated EAU mice. **(A)** Networks of the alteration of the intercellular interactions between eight different types of cells in the EAU mice/normal controls (EAU/normal) and prednisone-treated EAU mice/EAU mice (prednisone/EAU) comparison. The lines indicate intercellular communication, the thickness of which corresponded to the number of intercellular ligand-receptor pairs. Abbreviations for each type of cells are the same as that in **Figure 5**. **(B)** Network plots show the rescue effect of prednisone in terms of cell-cell communication, similar to **(A)**. Ligand-receptor pairs are rescued when they appear only in the EAU mice, not in the normal controls or prednisone-treated EAU mice. **(C)** Functional enrichment analysis of the rescued pairs. **(D)** The frequency (Freq) of rescued intercellular interaction pairs in eight types of cells. **(E)** Same as **(A)**, network plots indicate intercellular interaction alteration in 17 types of cells in EAU/normal or prednisone/EAU comparison. **(F)** Same as **(B)**, network plots show the rescue effect of prednisone in terms of cell-cell communication. **(G)** The frequency (Freq) of rescued intercellular interaction pairs in 17 types of cells.

The close communication between different types of immune cells forms the basis for effective immune responses, making it necessary to explore the intercellular interactions between immune cells. Thus, we explored the intercellular communication in EAU and upon prednisone treatment by determining the number of ligand-receptor pairs between different immune cell types and within the same cell type, indicating autocrine signaling (**Figure 6** and **Supplementary Figures 4C–E**).

We observed globally enhanced cell-cell communication in EAU mainly among myeloid cells, but not among T cells, probably because of the complex or even reversed transcriptional alterations in different T cell subsets (**Figure 6A**). Prednisone enhanced NK cell-associated communication and inhibited monocyte-associated communication (**Figure 6A**). The rescue effect, based on computational cell-cell communication, was primarily observed in macrophages, monocytes, and neutrophils (**Figure 6B**). We observed a strong impact of EAU and prednisone and a rescue effect on monocytes, macrophages, and neutrophils, indicating that these cells were highly sensitive to the changes in the immune system and presence of prednisone.

Then, we performed functional and pathway enrichment analyses on the rescued pairs to further examine the effects of prednisone. The erased pairs referred to the ligand-receptor pairs appear only in the EAU mice, not in the normal controls or prednisone-treated EAU mice (**Figure 6C**). Functionally, the eliminated interaction pairs were mostly associated with cell-cell adhesion and migration, inflammatory response, T cell activation and proliferation, and humoral immune response (**Figure 6C**). The most frequently rescued interaction pairs included the pro-inflammatory pair TNF-TNFRSF1A and B cell stimulatory pairs CD28-CD86 and CCL4-CCR5, which regulate immune cell migration (**Figure 6D**) (25, 26).

Furthermore, to better explore the changes in lymphocyte subsets, we reconstructed the cell-cell communication networks (**Figure 6E**). In EAU, the communication status of monocytes, macrophages, neutrophils, and T cells (Treg, Th17, and Mki67+ proliferating T cells) were significantly changed (**Figure 6E**). Prednisone seemed to have a complex influence on the communication networks, exhibiting both enhancing and inhibitory effects on all subsets (**Figure 6E**). As for the eliminated pairs, we observed widespread rescue effects on several CD4+ T cell subsets, including Treg, Th17, Tfh, Th1, and Mki67+ proliferating T cells (**Figure 6F**). The rescue effect on monocytes was also considerable, suggesting that monocytes may also play an important role in both the pathogenesis of uveitis and treatment with prednisone. In detail, eliminated interaction pairs that appeared frequently included CCL4-CCR5, CTLA4-CD86, CXCL10-CXCR3, and TNF-TNFRSF1 (**Figure 6G**), implying that prednisone may inhibit immune cell adhesion/migration, suppress inflammation, and control aberrant immune cell inactivation (25). Overall, abnormal intercellular communication patterns in EAU, especially those that mediate migration and inflammation, were shown to be alleviated by prednisone.

## Discussion

Here, we presented an integrative and comprehensive immune cell atlas of EAU and prednisone-treated EAU mice at the single-cell level. We studied the transcriptional changes in EAU in terms of cell type composition, gene regulation, transcriptional regulation, and intercellular communication, providing clues about the processes involved in EAU. Comparative analysis of the transcriptional features in EAU and prednisone-treated EAU mice revealed the underlying mechanisms by which prednisone achieves rapid autoimmune disease control. Our results depict the immune cell atlases in EAU and upon prednisone treatment and provide resources for further research.

In EAU, a classical model of autoimmune diseases within CNS, retinal autoantigens drive the development of retinal inflammation, which involves nearly all types of immune cells and multiple organs beyond the eye (12, 13, 24). High-dimensional assays and computational tools have allowed large-scale and comprehensive research on CNS autoimmune disease processes. Despite extensive investigation on CNS inflammatory diseases, including rheumatoid arthritis, multiple sclerosis, and experimental autoimmune encephalomyelitis, using bioinformatic methods, the transcriptional features of autoimmune uveitis have not been explored *via* high-throughput approaches (27). Moreover, a limited number of high-dimensional studies have focused on the EAU mouse model, which is the most important model for investigating the pathogenesis of uveitis and its associated therapies (13). Recently, several studies have revealed the transcriptional modifications in retinal endothelial cells and emphasized their potential roles in disease onset (28). However, most of these studies were based on bulk RNA sequencing and depicted only the average transcriptional changes in autoimmune states (24, 28–30). In the current study, we depicted lymph node immune modifications in EAU from multiple aspects. Consistent with the current understanding and clinical features of EAU, we found an increase in the proportions of Th1 and Th17 cell and a decrease in the proportion of Treg cells (31). Specifically, our results, which showed that Th1, Th17, and Treg cells were the top three most significantly modified T cells in EAU, were consistent with those of previous studies (24). Previous studies have indicated that monocytes/macrophages cause pathogenic consequences, while dendritic cells manage antigen presentation in autoimmune states (31–33). Similarly, our results showed that myeloid clusters, including monocytes, macrophages, and cDCs, were strongly affected in EAU and that cell-cell communication was globally enhanced, as the hypersensitive immune system was activated by autoantigens.

GCs are currently the most widely used immunosuppressive agents for the control of severe autoimmune diseases and are the first-line systemic treatment for patients with sight-threatening uveitis (10). Despite their extensive clinical application in autoimmune uveitis, the underlying mechanisms by which GCs repress inflammatory gene expression remain poorly understood.

Thus, we conducted a comparative analysis of the transcriptomes of EAU mice and prednisone-treated EAU mice (12, 13). In our data, we showed that prednisone may induce anti-inflammatory effects by regulating immune cell composition, particularly of Th1, Tfh, Th17, and Mki67+ proliferating T cells. Among all immune cell subpopulations distinguished in the lymph nodes, CD4+ T cell subsets (Th1, Treg, Tfh, and Th17 cells) and myeloid cells were most strongly subjected to the rescue effects or prednisone, suggesting that these cells were the main responders to prednisone treatment (34). GCs have been reported to downregulate the constituent transcription factor AP-1 components Jun and Fos and reduce the DNA-binding ability of the AP-1 components to their cognate DNA motifs (2). Hsp90, an important GC receptor chaperone protein, is thought to be the key regulator of GC effects (35). Consistently, we found commonly rescued AP-1 components and *Hsp90aa1* in more than four types of immune cells. Analysis of the transcriptional regulatory networks further supported these findings. In addition, we also found the rescued genes *Id3*, *Tspan32*, and *Vim* in more than four types of cells, extending the transcriptional effects of prednisone and EAU. These three genes have been reported to be involved in lymphocyte development, Th cell immune response regulation, and lymphocyte/neutrophil apoptosis, respectively (36–39). Based on computational transcriptional regulatory networks, the TFs Cebpb and Bhlhe40, which were rescued in two types of cells, may also play a central role in the rescue effects of GCs.

Interestingly, we also identified cell type-specific rescued genes, indicating the complex modifications to immune cell transcriptomes due to prednisone. Chemokine genes, including *Cxcl2* (neutrophils and monocytes), *Ccl3* (NK cells), and *Cxcl10* (monocytes), and chemokine receptors, particularly *Cxcr4* (T and B cells), were found to correlate with the role of prednisone in regulating immune cell migration (25). Additionally, *Hif1a*, which is linked to the induction of Foxp3 and promotion of Treg differentiation, was specifically rescued in T cells (40). In macrophages, *Irgm1*, which is related to the disruption of the blood-brain barrier in the initiation of inflammation, was specifically rescued (23). The rescue effects of prednisone were further depicted from the perspective of cell-cell communication, highlighting the important migration-managing roles of myeloid cell subsets (34, 41, 42). Our findings corresponded to those of previous studies, which demonstrated monocyte/macrophages as the main effector cells in cell mediator release and inflammatory infiltration.

Taken together, our results provide single-cell expression landmarks that aid in systematically annotating immune cell types, identifying sets of genes and key TFs that are differentially regulated in EAU and by prednisone, and constructing cell-cell communication networks modulated in EAU and by prednisone. Our data help elucidate the complex processes involved in EAU and the mechanisms of action of GCs.

## Data Availability Statement

The single-cell sequencing data have been deposited at GSA with the project number PRJCA005205 and GSA accession number HRA000850.

## Ethics Statement

The animal study was reviewed and approved by Ethics Committee of Zhongshan Ophthalmic Center, Sun Yat-Sen University.

## Author Contributions

WS designed the study. XW and HL conducted the experiment and acquired the data. RW analyzed the data and prepared the figures. YG, RD, and ZL wrote the manuscript. LX and LZ assisted the experiments. BC performed the statistical analyses. The manuscript was reviewed by all authors. Order of co-first author is based on the length of time spent on the project. All authors contributed to the article and approved the submitted version.

## Funding

This work was supported by the National Key Research and Development Program of China (2017YFA0105804).

## Conflict of Interest

The authors declare that the research was conducted in the absence of any commercial or financial relationships that could be construed as a potential conflict of interest.

The handling editor declared a shared affiliation with one of the authors (XW) at time of review.

## Publisher’s Note

All claims expressed in this article are solely those of the authors and do not necessarily represent those of their affiliated organizations, or those of the publisher, the editors and the reviewers. Any product that may be evaluated in this article, or claim that may be made by its manufacturer, is not guaranteed or endorsed by the publisher.
