# Supplementary figures and images for: Prednisone Reprograms the Transcriptional Immune Cell Landscape in CNS Autoimmune Disease
Source: Front Immunol. 2021 Aug 13;12:739605. doi: 10.3389/fimmu.2021.739605 (PMC8414592; doi:10.3389/fimmu.2021.739605)

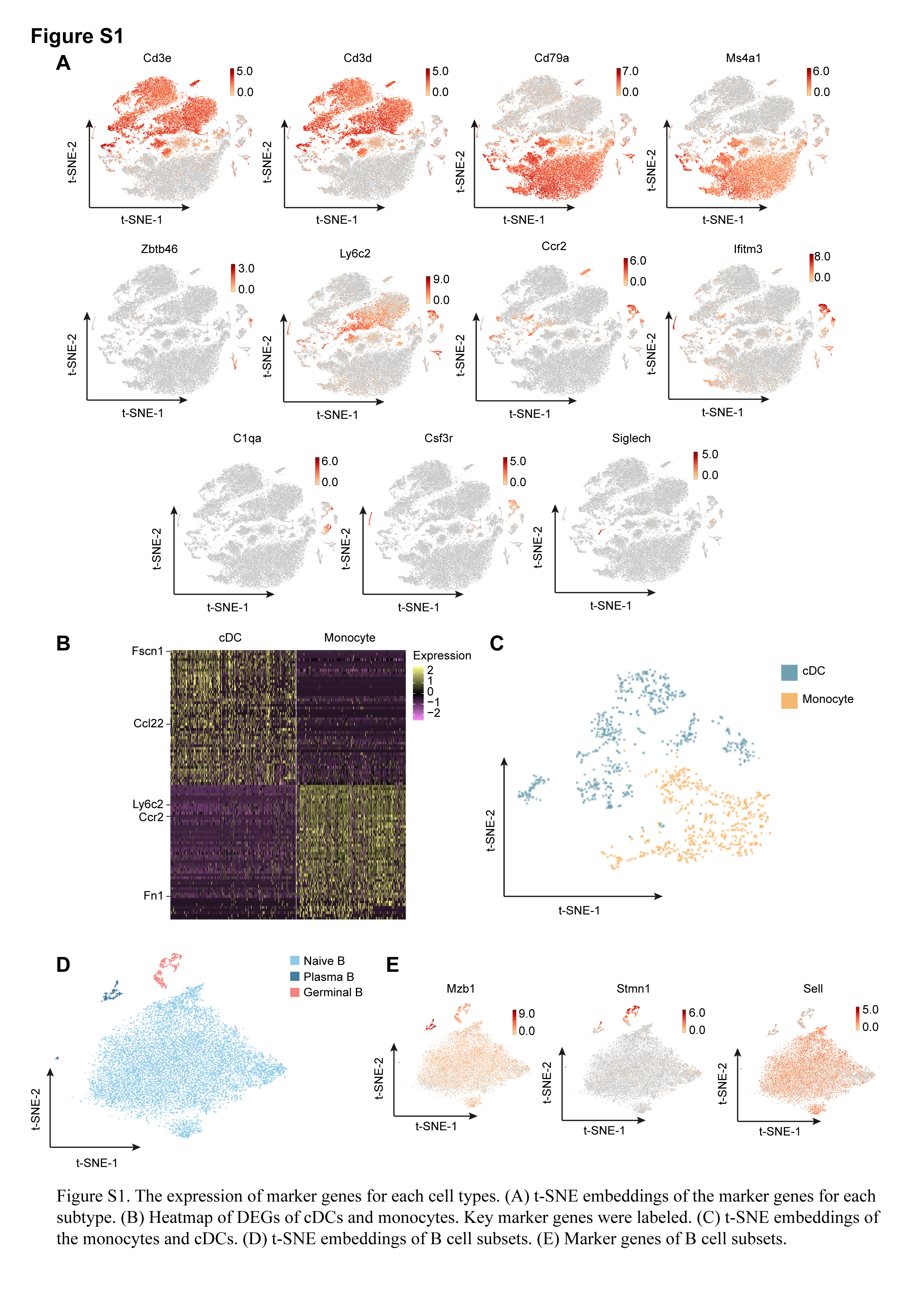

Supplement: Supplementary file 1 [file Image_1.tif]

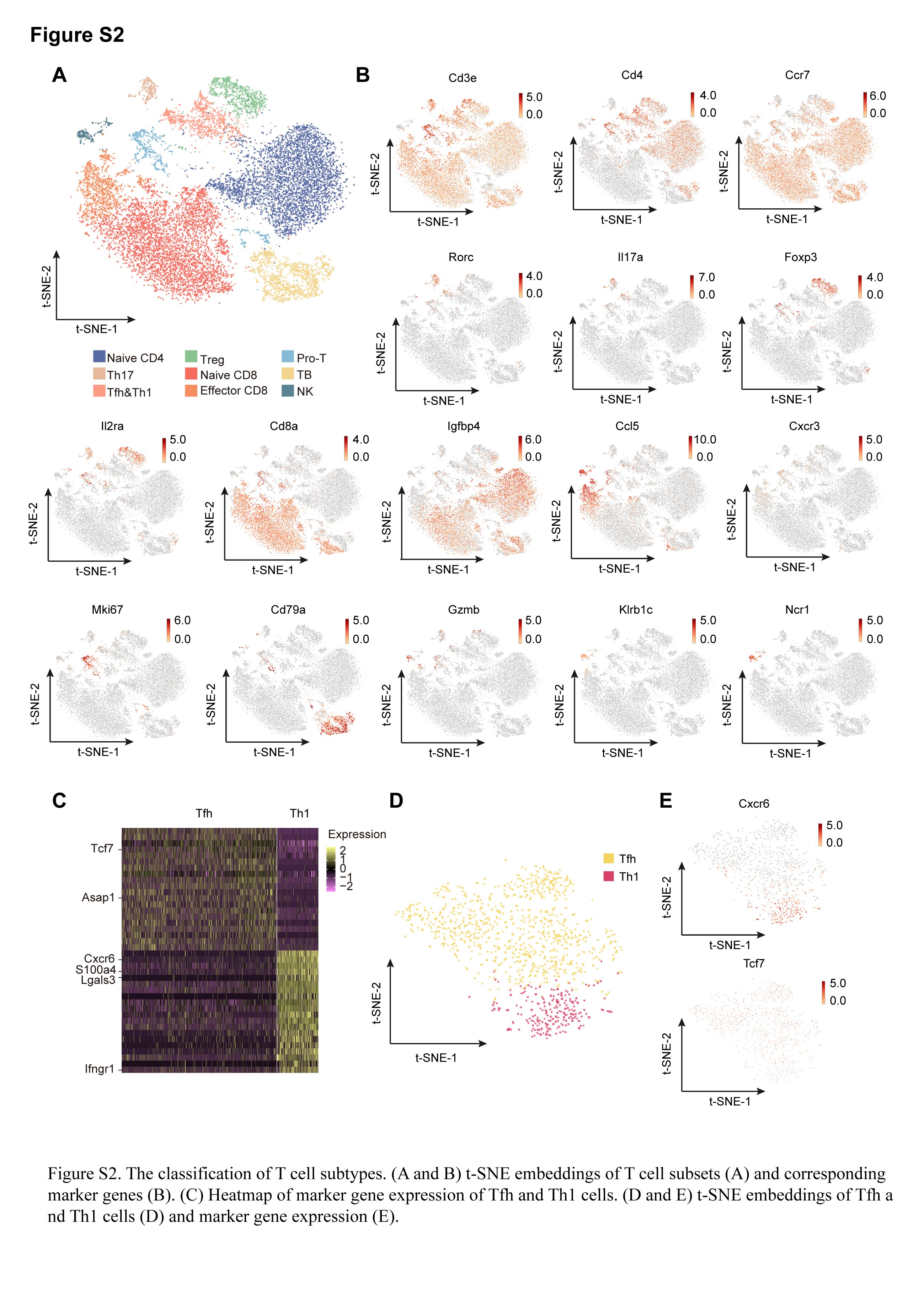

Supplement: Supplementary file 2 [file Image_2.tif]

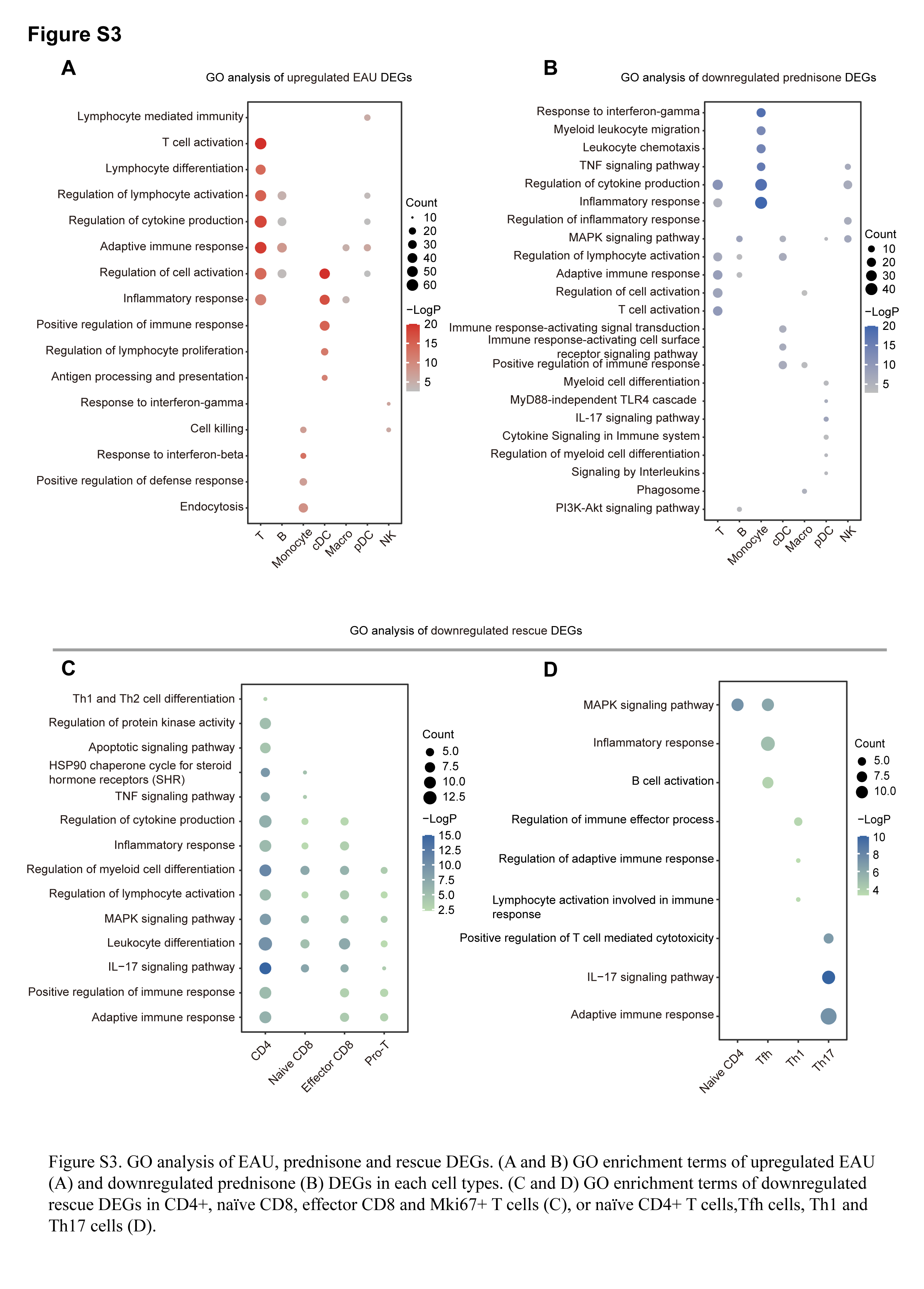

Supplement: Supplementary file 3 [file Image_3.tif]

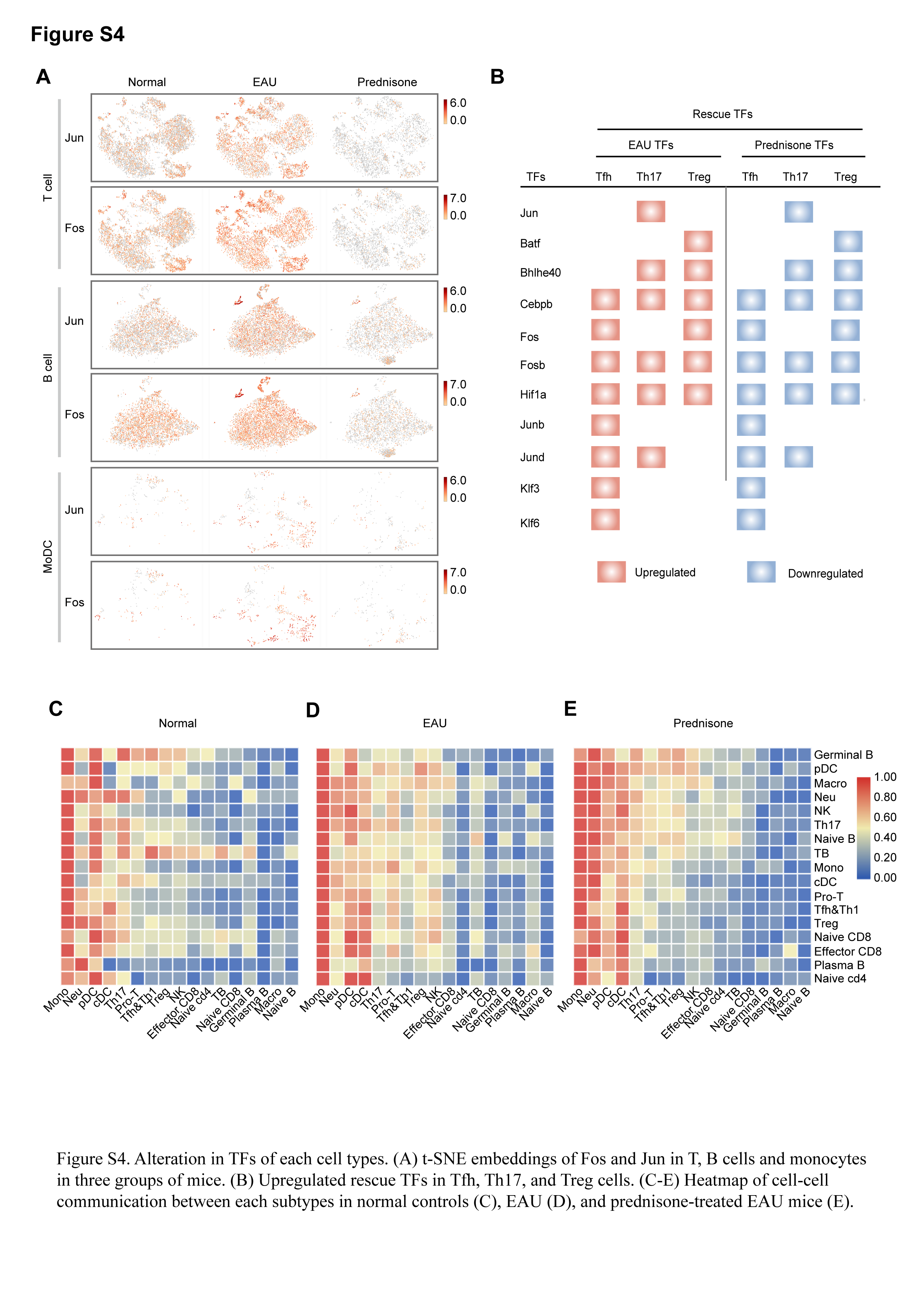

Supplement: Supplementary file 4 [file Image_4.tif]
